# Colchicine plus Dapsone in Colchicine‐Resistant FMF Patients

**DOI:** 10.1155/2019/2716127

**Published:** 2019-01-09

**Authors:** Farhad Salehzadeh, A. Enteshary, M. Moshkbar

**Affiliations:** ^1^Professor in Pediatric Rheumatology, Pediatric Department, Bouali Children's Hospital, Ardabil University of Medical Sciences (ARUMS), Ardabil, Iran; ^2^Assistant Professor of Rheumatology, Internal Medicine Department, Imam Khomeini Hospital, Ardabil University of Medical Sciences (ARUMS), Ardabil, Iran; ^3^Pediatric Department, Bouali Children's Hospital, Ardabil University of Medical Sciences (ARUMS), Ardabil, Iran

## Abstract

Five to ten percent of FMF patients have unfavorable response to the colchicine as a standard therapy. Biologic treatments have been shown to be highly effective, but there are often unavailable, because the price is unaffordably high. This study shows the striking effect of combined dapsone and colchicine therapy in such patients and recommends it as an alternative therapy in colchicine-resistant (CR) patients.

## 1. Introduction

Familial Mediterranean fever (FMF) is an auto-inflammatory monogenic disease that presents with recurrent painful attacks of fever, peritonitis, pleuritis and arthritis. In most patients, daily administration of colchicine can prevent a typical attack [1]. However, about five to ten percent of patients are completely resistant to colchicine or they have intolerable side effects due to this drug [2].

The therapeutic options for patients with colchicine‐resistant FMF (CR-FMF) patients are biologic drugs; although recent studies have shown their efficacy and positive results, availability and the cost of this modality are the main limiting factors [3, 4].

A previous study about dapsone in children with CR-FMF suggests favorable results [5]; consequently, this study has been planned to evaluate dapsone plus colchicine as a combination therapy in adult and adolescent CR-FMF patients.

## 2. Case Reports

We tried this combination therapy in 6 patients; all of them have been checked for the G6PD enzyme before treatment and filled consent confirmation. The study has been confirmed by the local ethical committee of faculty of medicine.

Case 1 is a 26‐year‐old female with negative history of FMF in her family. The first presentation of this disease started at the age of 14 including: abdominal pain, fever, nausea, arthralgia in knee joints, and erythematous swelling of the limbs (erysipelas‐like eruption). She usually had an attack every 2 weeks that would last for 1-2 days with a severity score of 10. Her *MEFV* gene mutation was: E148Q (heterozygous).

She started using colchicine 1 mg daily about 12 years ago but the results were not desirable enough, and she increased the dose to 2.5 mg daily, and then, the number of attacks was reduced to one attack every 20–30 days that would last 1 day with a severity of 5-6. We added dapsone 100 mg daily about 3.5 years ago, and since then, she did not have any similar attacks, except by discontinuation of the drug because of its unavailability in a short course period, her attacks recurred.

There have been no known side effects of colchicine or dapsone in this case.

Case 2 is a 40‐year‐old female with no history of FMF in her family. The onset of disease was from age 20 with abdominal pain, chills and fever, diarrhea, sweating, and arthralgia such as knee pain. She had an attack every 2 weeks that would last for 3-4 days with a high severity score near 10.

She started using colchicine 1 mg three times daily about 10 years ago, and then after the number of attacks was decreased to one episode every 2-3 months lasting for 1 day with a severity score of 7-8. Her MEFV gene mutations were M680I (G/C)/V726A (compound heterozygous).

From 9 months ago, we started using dapsone 50 mg daily, and since then, she was free of similar symptoms.

She suffers from occasional dizziness as known side effect of this drug.

Case 3 is a 38‐year‐old male with positive family history of FMF. He presented his first signs at the age of 14 but with delayed diagnosis until he was 30 years old. His symptoms were fever, arthralgia, body pain, and oral ulcer. The frequency of his attacks was variable, and each attack lasted for 3-4 days with a high severity score.

He started using colchicine 1 mg daily 8 years ago, and then after he suffered similar attacks with no response to colchicine although with high doses (2 mg). His *MEFV* gene mutations were C.1981G > T (D661Y), we submitted this as a new mutation in infevers.

From 9 months ago, we started dapsone 50 mg daily. Dapsone significantly controlled the attacks during this time. There have been no known side effects of this treatment in this case.

Case 4 is a 32‐year‐old male with negative family history of FMF. His first presentation was at the age of 23 years old with abdominal pain, vomiting, fever, chills, and limbs pain.

He was diagnosed with FMF at the age of 25, and his MEFV gene mutations were M680I (G/C) M680I (G/C) (homozygous). His attack intervals were almost 10 days, and it lasts for 3-4 days with a high severity score.

He started using colchicine 1 mg twice daily from 7 years ago, and since then, the number of attacks was decreased to one attack every month with similar pattern.

Seven months ago we added dapsone 50 mg daily, and since then, he was completely free of symptoms. There have been no known side effects of this therapy.

Case 5 is a 12‐year‐old female with negative family history. Her first signs presented at 3 years of age with: fever, abdominal pain, vomiting, chills, and sweating. She usually had an attack biweekly, lasting for 3-4 days with a high severity score. Her *MEFV* gene mutations were M694V/M680I (heterozygous).

She started using colchicine 1-2 mg daily from 6 years ago with unfavorable response. From 7 months ago, we added dapsone 50 mg daily, and since then, she has not have any attacks.

Case 6 is a 17‐year‐old female with negative family history. Her first sign was presented at one year of age with: fever and chills, nausea, and body pain with a high score. Her *MEFV* gene mutations were M694V (homozygous).

She started taking colchicine 0.25 mg daily 16 years ago then increased the dose to 2.5 mg daily; however, attacks persists to one episode every 1-2 months with similar severity.

From 7 months ago, we added dapsone 100 mg daily. Dapsone has reduced the frequency and severity of attacks (one attack in 5 months with a very low severity score). There have been no known side effects of this treatment in this case.

Table 1 shows these patients' data and summarized their findings.

## 3. Discussion

The exact mechanism of colchicine in controlling FMF attacks is not completely clear yet. Colchicine inhibits microtubule assembly in vitro conditions [6]. Microtubules are filamentous structures that help the maintenance of the structure of cells and are involved in cell movement for functions such as cytokine secretion, cell and nuclear divisions, and regulation of ion channels [7].

It is believed that the anti-inflammatory effect of colchicine in FMF is a result of microtubule disruption in neutrophils, which prevents their migration in response to chemotactic factors [8].

A new insight into colchicine efficacy is its effect in FMF through activation of the GTPase RhoA and subsequent phosphorylation of the 14-3-3 protein and inhibition of pyrin-induced inflammasome formation; Park et al. have found that colchicine resistance may also result when certain mutations in pyrin prevent binding to the 14-3-3 protein [9].

In review of the literature, there are some reports about alternative drugs especially biologics in CR-FMF patients. Intravenous colchicine 1 mg weekly in addition to the regular oral regime in patients suffering disabling attacks despite receiving maximal tolerated dose of oral colchicine may be beneficial [10].

In 2006, a young man with FMF and systemic amyloidosis who was not eligible for colchicine treatment because of gastrointestinal toxicity and ongoing dialysis was successfully treated with anakinra [11], and there is a report about a child who had frequent severe FMF attacks despite colchicine therapy was treated with this drug [12]. A small randomized placebo‐controlled trial showed that Rilonacept, an IL-1 inhibitor, significantly reduced the frequency of FMF attacks [13].

Canakinumab, a high-affinity human anti-IL1b monoclonal antibody [14], was used for treating two children with colchicine‐resistant FMF [15]. Recently, the efficacy of biologic drugs has been shown it has been shown an effective biologic drug in different auto-inflammatory diseases.

Two months of canakinumab therapy in another case, a 14-year-old female patient nonresponsive to colchicine and anakinra, resulted in remission of FMF attacks and improvement in inflammatory markers [17]. Several case reports have showed that TNF inhibitors such as etanercept and infliximab can be effective in controlling and improvement of FMF attacks [18–20].

The anti-inflammatory effect of dapsone is believed to be related to stabilization of neutrophil lysosomes [21]. Different studies showed that dapsone may impair neutrophil chemotaxis [22]. Dapsone could suppress integrin-mediated neutrophil adherence function. It also inhibits chemo attractant-induced signal transduction and thus suppresses neutrophil recruitment and local production of toxic products in the affected skin of neutrophilic dermatoses [23]. It can also suppress human neutrophil superoxide production and elastase release [24].

Therefore, it can be concluded that neutrophils and neutrophil products are the main targets of dapsone. patients' complete clinical response, lack of attacks by using a combination of two anti-inflammatory drugs was significant.

It seems that the synergetic effect of colchicine and dapsone in disturbing the neutrophils function as a combination therapy in controlling of FMF attacks in CR-FMF patients' emphasis this positive clinical response.

## 4. Conclusion and Recommendations

The combination of colchicine plus dapsone appears remarkably effective in controlling the attacks in CR-FMF patients, and our suggestion of a prospective controlled trial of this promising drug is warranted.

## Figures and Tables

**Table 1:** Details of patients' data.

				Before colchicine	After colchicine		After dapsone plus colchicine	
Patient no.	Age (years)	Sex	Duration (years)	Attack interval	Dosage of colchicine	Attack interval	Number of attacks	Attack interval
1	26	F	12	14 days	2.5 mg daily	20–30 days	0	—
2	40	F	20	14 days	3 mg daily	2-3 months	0	—
3	38	M	24	1-2 weeks	1-2 mg daily	1 month	0	—
4	32	M	9	10 days	2 mg daily	30 days	0	—
5	12	F	9	3-4 days	1-2 mg daily	2 weeks	0	—
6	17	F	15	15–20 days	2.5 mg daily	1-2 months	1	5 months
